# The Influence of Surgical Complexity and Center Experience on Postoperative Morbidity After Minimally Invasive Surgery in Gynecologic Oncology: Lessons Learned from the ROBOGYN-1004 Trial

**DOI:** 10.1245/s10434-024-15265-1

**Published:** 2024-04-15

**Authors:** Eric Lambaudie, Emilie Bogart, Marie-Cécile Le Deley, Houssein EL Hajj, Tristan Gauthier, Thomas Hebert, Pierre Collinet, Jean Marc Classe, Fabrice Lecuru, Stephanie Motton, Vanessa Conri, Catherine Ferrer, Frederic Marchal, Gwenael Ferron, Alicia Probst, Camille Jauffret, Fabrice Narducci

**Affiliations:** 1 Paoli Calmettes Institute, Marseille, France; 2 Oscar Lambret Cancer Center, Lille, France; 3 Université Paris-Sud, UVSQ, CESP, INSERM, Université Paris-Saclay, Villejuif, France; 4 University Hospital–Limoges, Limoges, France; 5 University Hospital–Tours, Tours, France; 6 University Hospital–Lille, Lille, France; 7 Institut de Cancérologie de l’Ouest–Nantes, Nantes, France; 8 Curie Institute, Paris, France; 9 University Hospital–Toulouse, Toulouse, France; 10 University Hospital–Bordeaux, Bordeaux, France; 11 University Hospital–Nîmes, Nîmes, France; 12 CRAN, UMR 7039, CNRS Institut de Cancérologie de Lorraine Vandoeuvre les–Nancy, Université de Lorraine, Nancy, France; 13 Institut Claudius Regaud Cancer Center–Toulouse, Toulouse, France

**Keywords:** Randomized phase III trial, Conventional laparoscopy, Robotic-assisted laparoscopy, Morbidity, Prognostics factors, Gynecologic oncology

## Abstract

**Background:**

This study was a secondary analysis of the ROBOGYN-1004 trial conducted between 2010 and 2015. The study aimed to identify factors that affect postoperative morbidity after either robot-assisted laparoscopy (RL) or conventional laparoscopy (CL) in gynecologic oncology.

**Methods:**

The study used two-level logistic regression analyses to evaluate the prognostic and predictive value of patient, surgery, and center characteristics in predicting severe postoperative morbidity 6 months after surgery.

**Results:**

This analysis included 368 patients. Severe morbidity occurred in 49 (28 %) of 176 patients who underwent RL versus 41 (21 %) of 192 patients who underwent CL (*p* = 0.15). In the multivariate analysis, after adjustment for the treatment group (RL vs CL), the risk of severe morbidity increased significantly for patients who had poorer performance status, with an odds ratio (OR) of 1.62 for the 1-point difference in the WHO performance score (95 % CI 1.06–2.47; *p* = 0.027) and according to the type of surgery (*p* < 0.001). A focus on complex surgical acts showed significant more morbidity in the RL group than in the CL group at the less experienced centers (OR, 3.31; 95 % CI 1.0–11; *p* = 0.05) compared with no impact at the experienced centers (OR, 0.87; 95 % CI 0.38–1.99; *p* = 0.75).

**Conclusion:**

The findings suggest that the center’s experience may have an impact on the risk of morbidity for patients undergoing complex robot-assisted surgical procedures.

**Supplementary Information:**

The online version contains supplementary material available at 10.1245/s10434-024-15265-1.

Robot-assisted laparoscopy (RL) in gynecologic oncologic surgery has been found equivalent to conventional laparoscopy (CL) in terms of various perioperative outcomes and offers additional benefits such as three-dimensional stereoscopic vision, wristed instrumentation, controlled tremor, and better ergonomics. However, the cost for this technology is higher than for laparoscopy, although it still is lower than for open surgery and could potentially be reduced with increased use.^1^ Despite a lack of data comparing RL and CL morbidity, most retrospective studies have concluded that the morbidity of RL is equal to that of CL or better.^2–4^

The ROBOGYN-1004 randomized controlled trial (NCT01247779)^5^ compared the severe peri- and postoperative morbidity in patients managed for gynecologic cancers with RL versus CL and concluded that RL is not superior to CL regarding the incidence of severe postoperative morbidity. Overall, we observed a nonsignificant increase in the rate of severe morbidity in RL versus CL groups (Relative Risk of Robotic Assisted Laparoscopy/Conventional Laparoscopy, 1.30; 95% confidence interval CI 0.90–1.87; *p* = 0.15). Similar to the findings in the updated, merged review of the two Cochrane reviews evaluating RL in gynecologic surgery and in the gynecologic oncology,^6^ the ROBOGYN-1004 trial also concluded that RL is associated with a significantly longer operating time than CL (190 vs 145 min), but no difference was observed in the rate for conversion to another surgical approach. These findings may be influenced by different factors. Patient characteristics, comorbidities, and the complexity of the surgical procedures may constitute independent factors that increase the technical difficulties and may act as confounding factors in evaluating the risk of severe postoperative morbidity.

The difference in skill progression between the two techniques is another important factor to consider. Studies have shown that to reach a plateau in operative time or reduce postoperative morbidity, it may take 25–50 surgeries using RL for gynecologic cancers.^7,8^ Furthermore, the variation in skills and proficiencies of the surgeons in randomized controlled trials remains a challenge, as discussed in the Laparoscopic Approach to Cervical Cancer (LACC) trial.^9^

Thus, the objective of this post hoc analysis was to identify the prognostic factors associated with postoperative morbidity at 6 months, considering the patient characteristics, the surgical characteristics, and the center’s characteristics, as well as the surgeons’ skill progression.

## Methods

### Trial Design

The ROBOGYN-1004 was a multicenter, randomized phase 3 trial that compared the safety and efficacy of RL and CL for patients with gynecologic cancer requiring minimally invasive surgery (MIS). The trial was conducted in 10 French centers between December 2010 and November 2015.

The ROBOGYN-1004 trial included patients with a diagnosis of gynecologic cancers planned for whom MIS. Eligible patients included those with endometrial cancer who underwent hysterectomy with or without pelvic lymphadenectomy, patients with early cervical cancer who underwent radical hysterectomy with or without pelvic lymphadenectomy, patients with locally advanced cervical cancer who underwent para-aortic lymphadenectomy tailoring chemoradiation or surgery after concomitant chemoradiation, and patients who had early ovarian cancer with lymph node restaging.

The surgical approach was randomized before surgery, balanced 1:1 and stratified by center. Patients, physicians, and investigators were aware of the assigned treatment group before surgery. However, the independent expert surgeons’ review committee was blinded to group allocation. All the patients in the primary analysis were included in the current study.

#### Treatment and Monitoring

In this study, the surgical procedures were similar for RL and CL. The Da Vinci system (Intuitive Surgical, Sunnyvale, CA) was used for RL (Online Appendix). The participating centers had surgeons experienced in both RL and CL, with at least 20 RL procedures performed at each center before the trial.

Follow-up visits were planned to occur 2 and 6 months after surgery, then every 6 months for 2 years thereafter. Oncologic follow-up evaluation included gynecologic exams, biomarker assessments if applicable, magnetic resonance imaging (MRI) for cervical and uterine cancers, and computed tomography (CT) scans for ovarian cancers.

#### End Points Definition

The primary end point of the study was severe peri- or postoperative morbidity occurring during surgery or within 6 months afterward, as per the trial protocol.^5^

Intraoperative complications were graded according to the OSLO classification, (severe: grades ≥ 2).^10^ Severe early postoperative complications were defined as those occurring within 30 days after surgery with a Clavien-Dindo grade of 2 or higher.^11^ Severe late postoperative complications were defined as those occurring between 30 days and 6 months after surgery graded as 3 or higher according to NCI-CTCAE-v4.0.^12^ All severe complications were reviewed by a committee of independent expert surgeons blinded to group allocation.

We performed two sensitivity analyses similar to the primary analysis evaluating the proportion of patients experiencing any complications grade II/2 or higher (independently of the grading scale), and any complications grade III/3 or higher.

The following factors were evaluated for their prognostic and predictive value regarding the risk of morbidity:

### Evaluated Factors

#### Patient Characteristics

The patient characteristics included age at study entry, body mass index (BMI) at study entry (normal BMI [< 25 kg/m^2^] vs overweight [25–30 kg/m^2^] vs obesity [> 30 kg/m^2^]), World Health Organization (WHO) performance status studied as a continuous variable, surgical history, prior radiotherapy treatment, and tumor site (endometrial vs cervical cancer). Ovarian cancer cases were excluded in the evaluation of the association between tumor site and morbidity due to the very small number of patients.

#### Surgery Characteristics Led to Four Categories

The four categories were total hysterectomy with no lymph node dissection (TH alone), pelvic lymph node dissection with or without total hysterectomy (PeLND ± TH), aortic lymph node dissection with or without pelvic lymph node dissection, and with or without total hysterectomy but no radical hysterectomy (AoLND ± TH), radical hysterectomy with or without lymphadenectomy (RH ± LND) (Appendix Table S1).

#### Center Characteristics

The center’s experience with RL before the start of the trial was based on the number of RL procedures performed at each center before the first inclusion in the trial (< 50 vs ≥ 50).

To evaluate the skill progression, for each center, we divided the accrual period into two halves and considered the first half as the learning period.

### Statistical Considerations

The trial sample size of the ROBOGYN-1004 randomized trial was calculated expecting a 10% decrease in the proportion of patients with severe postoperative morbidity between the treatment groups (RL [5%] vs CL [15%]). To achieve a power of 90% and a two-sided alpha error of 5%, 374 patients were required

The current analysis was performed as a post hoc analysis after publication of the results from the randomized trial. A specific statistical analysis plan was defined before the start of these analyses.

The as-treated population was considered evaluable for safety. The analysis excluded patients who withdrew consent, those who did not undergo surgery, and those for whom a laparotomy was decided before surgery. For the patients who underwent surgery, the study accounted for the type of surgery (CL vs RL) actually performed, including a few patients whose treatment was switched to the other treatment before surgery.

Considering the number of patients evaluable for the primary end point (*n* = 368) and the observed proportion of severe postoperative morbidity at 6 months (overall: 24% [90/368]), the power of the current analysis was 92% to conclude a significant prognostic value for a binary factor equally distributed, associated with a 15 % difference in morbidity probability (16.5% vs 31.5%), with a two-sided alpha level of 0.05.

We evaluated the prognostic value of the different predictors by estimating odds ratios (ORs) in a two-level logistic regression analysis modeling the probability of postoperative morbidity and considering the center effect as a random effect to take into account patients in centers as clusters of observations.^13^ We first studied each factor separately in this hierarchical model (univariate analysis). We then performed multivariable analyses after selecting variables associated with a *p* value lower than 0.20 in the univariate analysis and checking the potential association between the different predictors within each level (patient and surgery characteristics at the patient level, center characteristics for the second level).

The final multivariate prognostic model included only covariates associated with a *p* value of 0.05 or lower, apart from the treatment effect (RL vs CL) that remained in the model regardless of the *p* value. We evaluated the stability of the final prognostic model in the sensitivity analyses considering GII/2+ morbidity (first sensitivity analysis) and GIII/3+ morbidities (second sensitivity analysis).

We evaluated the heterogeneity of the treatment effect (RL vs CL) on the risk of severe morbidity according to patient, surgery, and center characteristics in multivariable two-level logistic regression models, including the treatment effect and the variables of the final prognostic model plus each of the covariates successively, with an interaction term between the treatment effect and the studied covariate. For each covariate considered successively, the OR associated with the treatment effect was estimated by subgroup, and the heterogeneity of the treatment effect according to the covariate was tested by the interaction test, considering *p* values of 0.05 or lower as significant. The results of these heterogeneity analyses were illustrated using a forest plot. All estimates are provided with their 95% confidence intervals (CIs).

This analysis was performed considering the primary definition of severe morbidity and GII/2+ morbidity (first sensitivity analysis). It could not be studied for GIII/3+ morbidity due to the small number of events.

It was anticipated that the risk of morbidity associated with RL could vary according to the center's experience when surgical procedures were deemed more complicated (i.e., when an aortic lymphadenectomy or a radical hysterectomy was performed). In contrast, no difference was expected in other simpler surgical procedures.^8^ Consequently, we also performed a subgroup analysis to evaluate the heterogeneity of treatment effect between more experienced and less experienced centers separately for patients undergoing an aortic lymphadenectomy or radical hysterectomy (AoLND ± TH or RH ± LND, classified as “difficult surgery”) and for the other patients (TH alone or PeLND ± TH, classified as “simple surgery”).

All statistical analyses were performed using Stata software, version 15.0 (StataCorp LLC, College Station, TX, USA).

## Results

### Description of the Study Population

**Fig. 1:**
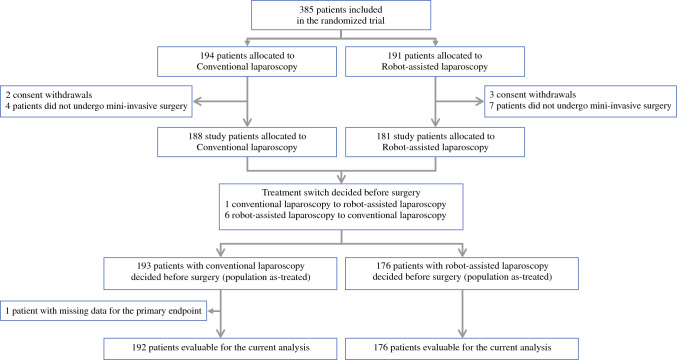
Study participant flow

Overall, 368 of the 385 patients in ROBOGYN-1004 trial were deemed evaluable for the analysis of severe morbidity and consequently included in the current analysis. Of the 368 patients, 176 underwent RL, and 192 underwent CL (Fig. 1). The median patient age was 59 years (range, 24–85 years). Table 1 summarizes patient and disease characteristics as well as the surgery and center characteristics. The characteristics according to the treatment group were published previously.^5^

**Table 1:** Characteristics of patient, disease, surgery, and centers, and analysis of association with the risk of severe morbidity (main analysis: *n* = 368)

Characteristics	Description		Univariate analysis^a^			Multivariable analysis^b^		
	*n/N*	%	OR	95 % CI	*p* Value	OR	95 % CI	*p* Value
*Treatment arm*								
Conventional laparoscopy	41/192	21	1	–	0.17	1		0.33
Robot-assisted laparoscopy	49/176	28	1.39	0.86–2.24		1.28	0.78–2.12	
*Patient characteristics*								
*Age at study entry*					0.44	–		
OR for 1-year difference			0.99	0.97–1.01				
*BMI (kg/m*^*2*^*)*					0.77			
< 25	45/170	26	1	–		–		
25–30	17/79	22	0.79	0.41–1.50		–		
> 30	28/119	24	0.93	0.52–1.66		–		
*WHO performance status*					0.077			0.027
OR for a 1-point difference			1.44	0.96–2.16		1.62	1.06–2.47	
*Prior surgery*					0.98			
No	79/320	25	1	–		–		
Yes	11/45	23	0.99	0.47–2.07		–		
*Prior radiotherapy*					0.41			
No	77/320	24	1	–		–		
Yes	13/48	27	1.37	0.65–2.88		–		
*Tumor site*					0.014			NS^c^
Endometrial cancer	40/199	20	1					
Cervical cancer	48/157	31	1.89	1.13–3.15				
*Surgery characteristics*								
*Type of surgery*^d^					<0.001			<0.001
TH alone	18/122	15	1	–		1		
PeLND ± TH	15/83	18	1.41	0.64–3.08		1.61	0.72–3.62	
AoLND ± TH	20/68	25	2.10	0.98–450		2.42	1.10–5.32	
RH ± LND	37/94	39	4.14	2.06–8.33		4.83	2.34–9.98	
*Center characteristics*								
*Center’s prior RL experience*^e^					0.99			
< 50 RL before start of inclusion	29/118	25	1			–		
≥ 50 RL before start of inclusion	61/249	25	1.00	0.60–1.66		–		
*Skill progression*								
*Period of accrual*					0.14			NS^f^
First half of accrual period	52/187	28	1					
Second half of accrual period	38/181	21	0.69	0.43–1.12				

*n/N*, no. of patients experiencing a severe morbidity/no. of patients in the subgroup; OR, odds ratio; CI, confidence interval; BMI, body mass index; WHO, World Health Organization; TH, total hysterectomy; PeLND, pelvic lymph node dissection; AoLND, aortic lymph node dissection; RH, radical hysterectomy; LND, lymph node dissection; RL, robot-assisted laparoscopy

^a^To control for a possible center effect, hierarchical models were used, considering the effect center as a random effect for all analyses (uni- and multivariate analyses) except when the association between center characteristics and risk of morbidity was specifically studied. For these latter analyses, in the univariate analysis, each characteristic was considered as a fixed factor in the logistic regression model with no random center effect. None of the studied center characteristics was found significantly associated with the risk of morbidity.

^b^RL experience: robot-assisted experience, evaluated by the number of robot-assisted laparoscopies performed before accrual of the first patient in the trial. This information is missing for one center that recruited only one patient.

^c^Adjusted on the multivariable model, the tumor site appears not significantly associated with the risk of severe morbidity: OR cervix/endometrium = 1.24 (0.66–2.34); *p* = 0.51

^d^The definition of the categories of type of surgery is detailed in Appendix Table S1.

^e^The multivariable model includes the treatment effect (RL vs CL), and the two factors significantly associated with the risk of severe morbidity in the multivariable analysis (*p* < 0.05): WHO performance status and type of surgery, considering the center as a random effect.

^f^Adjusted on the multivariable model, the period of accrual appears not significantly associated with the risk of severe morbidity: OR second half/first half of accrual period = 0.71 (0.43–1.18); *p* = 0.19

The most frequently performed procedures were total hysterectomy (TH: *n* = 206, 56%) and radical hysterectomy (RH: *n* = 94, 26%). Lymphadenectomy was performed for 208 patients (57%). Pelvic lymph node dissection (PeLND) was performed for 168 patients and aortic lymph node dissection (AoLND) for 71 patients. As detailed in Appendix Table S1, this led to four categories denoting the burden of the surgical procedure: TH alone (*n* = 122), PeLND with or without TH (*n* = 83), AoLND with or without TH (*n* = 68), and RH with or without LND (*n* = 94). We did not observe any significant difference in the type of surgical procedure between the treatment groups.

Overall, 249 patients (69%) were recruited in centers that had RL experience exceeding 50 RL procedures before the start of the study, with a balanced distribution between treatment groups (120 RLs vs 129 CLs). The remaining 118 patients were recruited in centers with less experience (55 RLs and 63 CLs).

### Description of the Observed Intra- and Postoperative Morbidity

Severe intraoperative morbidity was experienced by 15 patients (9%) in the RL group and 7 patients (4%) in the CL group. Severe postoperative morbidity was experienced by 32 patients (17%) in the RL group and 34 patients (20%) in the CL group within 30 days after surgery and by 7 patients (4%) in the RL group and 8 patients (5%) in the CL group more than 30 days after surgery. Details of the intra- and postoperative morbidities are described in Appendix Table S2.

### Prognostic Value of Patient, Surgery, and Center Characteristics for Morbidity

Table 1 shows the prognostic analysis of patient, surgery, and center characteristics in severe postoperative morbidity. We observed a significantly increased risk of severe morbidity for patients with poor performance status (OR, 1.62), for a 1-point difference in WHO score (95% CI 1.06–2.47; *p* = 0.027) in the multivariable analysis with adjustment for type of surgery and treatment group (RL vs CL) and with control for the center effect. The risk also differed significantly according to the type of surgery between the patients who underwent TH alone (OR, 1.61; 95% CI 0.72–3.62), those who had PeLND ± TH), (OR, 2.42; 95% CI 1.10–5.32), those who had AoLND ± TH (OR, 4.83; 95% CI 2.34–9.98), and those who had RH ± LND), with a *p* value lower than 0.001 for the overall comparison.

The risk associated with the tumor site (cervix vs endometrium) in the univariate analysis (*p* = 0.014) was no longer significant in the multivariable model (OR, 1.24; 95% CI 0.66–2.34; *p* = 0.51). We observed no significant association with age (*p* = 0.44), BMI (*p* = 0.77), previous surgeries (*p* = 0.98), or previous radiation therapy (*p* = 0.41). Overall, the risk of severe morbidity did not differ significantly between the treatment centers, neither when the individual centers themselves were considered (*p* = 0.19) nor when the center’s prior RL experience was considered (*p* = 0.99).

Concerning the center’s experience during the study period, we observed slightly fewer complications during the second part of the accrual period than during the first period. However, this difference was not significant, neither in the univariate analysis (OR = 0.69; 95% CI 0.43–1.12; *p* = 0.14) nor in the multivariable analysis (OR, 0.71; 95% CI 0.43–1.18; *p* = 0.19).

### Effect of Robot-Assisted Laparoscopy Versus Conventional Laparoscopy

As previously described, we observed slightly more severe morbidity in the RL group (28%) than in the CL group (21%). However, the difference was not significant, neither in the univariate analysis (OR, 1.39; 95% CI 0.86–2.24; *p* = 0.17) nor in the multivariable analysis, with control for patient performance status and type of surgery (OR, 1.28; 95% CI 0.78–2.12; *p* = 0.33).

In the multivariable sensitivity analyses, the difference between RL and CL appeared larger, although the difference was not significant (OR, 1.51; 95% CI 0.93–2.43; *p* = 0.097 when complications grade 2/II or higher were considered, and OR, 1.95; 95% CI 0.94–4.02; *p* = 0.071 when complications grade 3/III or higher were considered) (Appendix Table S3).

### Treatment Effect Heterogeneity According to the Studied Factors

**Fig. 2:**
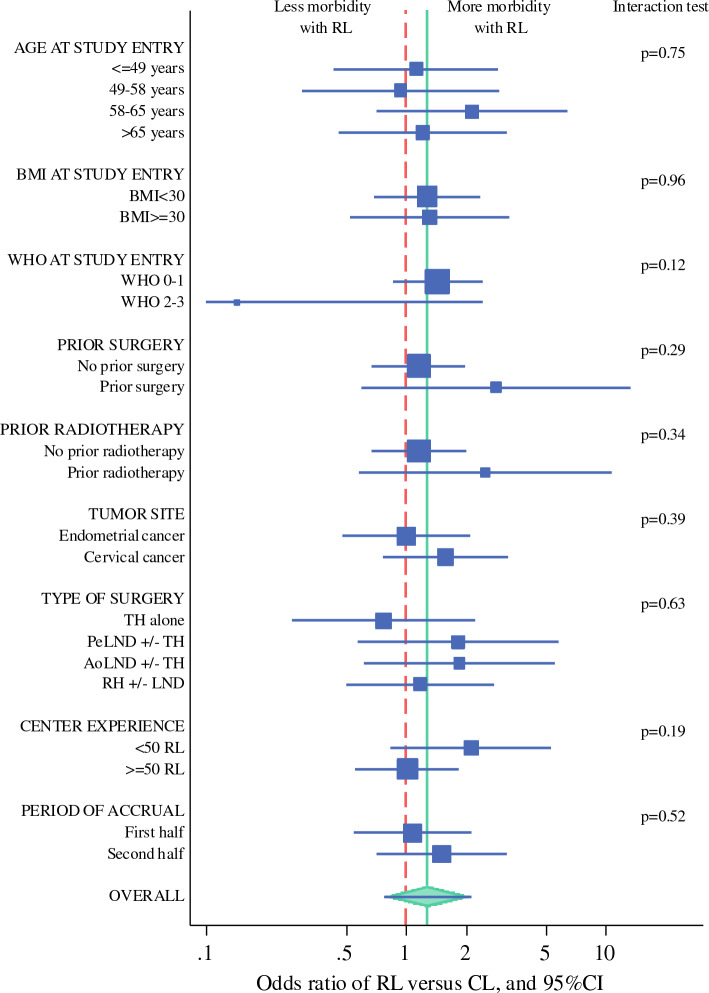
Forest plot of the treatment effect on severe postoperative morbidity by subgroup (main analysis). The lower boundary of the 95% confidence interval of WHO 2– is not represented because of the scale. The 95% confidence interval (CI) is 0.01–2.42. For each factor successively, the treatment effect of RL versus CL in the different subgroups was estimated in a multivariable model, including WHO performance status, type of surgery, treatment arm, the considered covariate, and an interaction term between treatment and the covariate. All models were hierarchical, considering the center as a random effect, except when the treatment effect was studied according to prior center RL experience (no random center effect). For each factor successively, the *p* value corresponds to the interaction test of the treatment effect (RL vs CL) by the considered factor. Marker size is scaled according to the number of patients in each subgroup. Detailed results of this analysis are available in Appendix Table S4. BMI, body mass index; WHO, World Health Organization performance status; TH, total hysterectomy; LND, lymphadenectomy; PeLND, pelvic lymph node dissection; AoLND, aortic lymph node dissection; RH, radical hysterectomy; RL, robot-assisted laparoscopy; CL, conventional laparoscopy

Overall, the relative treatment effect of RL versus CL appeared relatively homogeneous across subgroups, as illustrated in Fig. 2 and Appendix Table S4. We observed a nonsignificant trend for a difference in treatment effect between patients with WHO scores 0 or 1 and those with WHO scores 2 or 3. Hence, in contrast to the increased risk associated with RL versus CL for patients with a WHO score of 0 or 1 (OR [RL/CL], 1.44; 95% CI 0.86–2.42), the risk associated with RL versus CL appeared lower in the small group of 12 patients with a WHO score of 2 or 3 (OR [RL/CL], 0.14; 95% CI 0.01–2.42). However, the *p* value of the interaction test was 0.12. The excess of morbidity associated with RL versus CL appeared larger in the centers that performed fewer than 50 robot-assisted laparoscopies before the first inclusion in the ROBOGYN-1004 trial (OR [RL/CL], 2.13; 95% CI 0.84–5.37) than in the more experienced centers (OR [RL/CL], 1.02; 95% CI 0.56–1.85). However, the interaction test was not significant (*p* = 0.19). For complication grades II+/2+, the results were relatively similar (sensitivity analysis is detailed in Appendix Table S4).

**Fig. 3:**
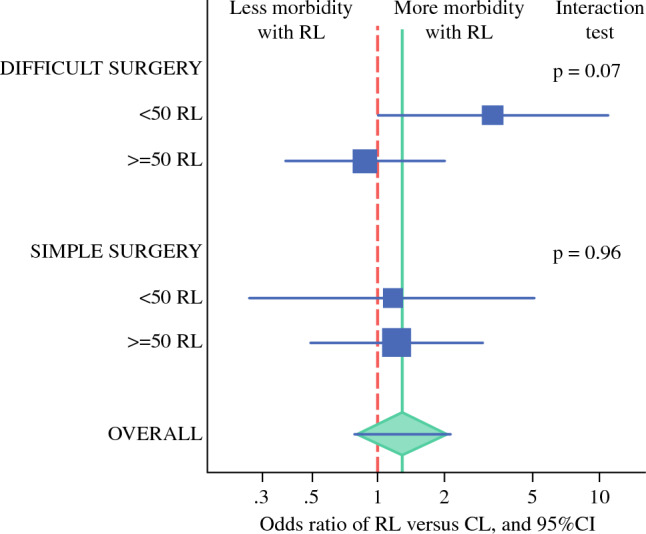
Forest plot of the treatment effect on severe postoperative morbidity according to the center experience, with simple and complex surgical acts considered separately. Treatment effect of RL versus CL was estimated by subgroups (centers with < 50 vs ≥ 50 robot-assisted laparoscopies before the first inclusion in the trial) in multivariable models, including the treatment arm, WHO performance status, prior center experience in robot-assisted laparoscopy, and an interaction term between treatment group and prior center RL experience, separately in the strata of simple surgical acts (TH alone or PeLND ± TH) and the strata of difficult surgical acts (AoLND ± TH or RH ± LND). The *p* value corresponds to the interaction test of the treatment effect (RL vs CL) and the center experience separately for (1) surgical acts classified as “simple surgery” (i.e., TH alone or PeLND ± TH) and (2) surgical acts classified as “difficult surgery” (i.e., AoLND ± TH or RH ± LND). Marker size is scaled according to the number of patients in each subgroup. Detailed results of this analysis are available in Appendix Table S5. RL, robot-assisted laparoscopy; CL, conventional laparoscopy; WHO, World Health Organization; TH, total hysterectomy; PeLND, pelvic lymph node dissection; AoLND, aortic lymph node dissection; RH, radical hysterectomy

As illustrated in Fig. 3 and detailed in Appendix Table S5, when simple surgical procedures (TH alone or PeLND ± TH) and difficult surgical acts (AoLND ± TH or RH ± LND) were considered separately, it appeared that the treatment effect was quite similar between centers with less experience and those with more experience when the surgery was simple (interaction test, *p* = 0.96). In contrast, we observed a statistically nonsignificant interaction between treatment effect and center experience in the difficult procedures (interaction test, *p* = 0.07). Hence, we observed an excess of morbidity after difficult procedures with RL versus CL in the less experienced centers (OR, 3.31; 95% CI 1.0–11; *p* = 0.050), indicating a trend toward statistical significance. In contrast, no such excess was observed in the experienced centers (OR, 0.87; 95% CI 0.38–1.99; *p* = 0.75).

## Discussion

Literature review has documented the advantages of MIS over laparotomy, particularly in endometrial cancer.^14^ Additionally, robotic assistance, which provides benefits such as enhanced precision and ergonomics, has led to increased adoption of robotic laparoscopy despite the absence of prospective studies assessing its outcomes.

Based on the ROBOGYN-1004^5^ results, the adoption of costly surgical systems, such as RL, should be approached with caution, particularly in the absence of robust scientific validation. Currently, tumor location, patient characteristics, surgical indication, and complexity are crucial factors when clinicians are deciding on a surgical approach.^6,15,16^

Our results showed that the risk of severe morbidity related to the tumor site (cervix vs endometrium) was significant in the univariate analysis, but no longer significant in the multivariable model. We observed no significant interaction between surgery method and tumor site. These results suggest the possibility of managing cervical or endometrial cancer with either RL or CL. Other factors not evaluated in the current analysis but also important to take into consideration include the previous treatments (e.g., brachytherapy and external beam radiation therapy).^17^

Our results showed a significant increase in the risk of severe morbidity among those with lower performance scores. This aligns with the results by Aloisi et al.^18^

Furthermore, we observed that the complexity of surgery was an independent predictor of complications for patients undergoing PeLND ± TH (OR, 1.61) those undergoing AoLND ± TH (OR, 2.42), and those undergoing RH ± LND (OR, 4.83) compared with simple hysterectomy. However, Narducci et al.^5^ described a significant reduction in the risk of urinary tract complications for patients treated with RL (2%) versus CL (7 %) (*p* = 0.04). These findings suggest that RL may confer an advantage in mitigating the risk of urinary tract complications, particularly in the setting of radical hysterectomy.

The impact that the complexity of surgery has on the rate of complications also was demonstrated in several studies evaluating patients undergoing ovarian cancer cytoreductions.^19–21^ In the SCROPION clinical trial, Fagotti et al.^22,23^ calculated a rate of 25.9% of severe complications for patients managed with frontline surgery for advanced-stage ovarian cancer, with most patients (89.3%) requiring high-complexity surgeries (Aletti score > 7) versus 7.6% of patients who underwent interval cytoreduction, with most patients (85.1%) requiring procedures with low to intermediate complexity (Aletti score ≤ 7) (*p* < 0.0001.

During the past decades, we witnessed a gradual development of MIS in gynecologic oncology. Despite this progress, the introduction of robotic assistance has contributed significantly to an increase in the adoption of MIS for even the most complex cases. However, the indications for robotic assistance have expanded rapidly, particularly for cervical cancer surgery, despite a lack of high-quality evidence supporting this new technology. In endometrial cancer, although the LAP-2 study^14^ compared CL with laparotomy, no comparable study has been conducted for robotic assistance.

Despite the technical advantages of robotic assistance, the acquisition of the required skills cannot be overlooked, especially when combined with a challenging procedure such as radical hysterectomy. The RL procedure involves multiple technical aspects including the handling of the patient’s cart, the surgeon’s console, hands and feet coordination, and management of the surgical team. Therefore, it is essential for the surgeons to acquire and validate a learning curve. Failure to do so may potentially increase the risk of intra- and postoperative complications, and also may have a negative impact on the oncologic outcomes. However, it is important to note that the exact impact of this learning curve on outcomes remains unknown at this point, and further studies are needed to provide a conclusive answer. One such ongoing study that may shed the light on this question is the Robotic Versus Open Hysterectomy Surgery in Cervix Cancer (ROCC) trial.^24^

No significant difference was found when the risk of severe perioperative morbidity was evaluated between the two groups. The treatment effect also was similar between centers with less and those with more experience for simple surgeries (interaction test, *p* = 0.96). However, we observed a trend toward an impact of center experience on treatment effect in the case of difficult procedures (interaction test, *p* = 0.07). Hence, we observed a borderline significant excess of morbidity after difficult procedures with RL versus CL in the less experienced centers (OR, 3.31; 95% CI 1.0–11) compared with no excess in the experienced centers (OR, 0.87; 95% CI 0.38–1.99). In fact, in the ROBOGYN-1004 trial, the recent implementation of RL in several centers was associated with a shorter learning curve, particularly for complex surgical procedures.

In their study evaluating the learning curve of RL in gynecologic oncology for both senior and junior surgeons, Jauffret et al.^8^ concluded that 50 procedures were necessary for a decrease in the complication rate. But these results may suggest that if surgical procedures are easier using the robotic assistance, a dedicated learning curve must be followed to limit the risk of complications.^25^ Recommendations have been published recently by the Society of European Robotic Gynaecological Surgery (SERGS).^26^

However, our study had some limitations. Although the participating surgeons were skilled at conventional surgery and had completed at least 20 surgeries by RL, their robot-assisted surgery experience may have been insufficient. Although this study provided insights into the prognostic factors at a center level, it did not disaggregate the surgical outcomes based on individual surgeon experience. Acknowledging this limitation, future studies could be designed to specifically explore the impact of individual surgeon experience on postoperative morbidity, offering a more granular understanding of the interplay between surgeon skill and patient outcomes.

In addition, because robot-assisted surgery was a new approach, surgeons may have been more exhaustive in reporting morbidities in this treatment group. Furthermore, the relatively small sample size of the study population was another limitation. It was defined to ensure a power of 90% for a 10% target difference in severe perioperative morbidity between RL and CL. This study had good power for analyzing major prognostic factors. However, it lacked power when analyzing the minor associations and the interactions between treatment effect and covariates, especially in small subgroups.

One also can regret the chosen primary end point of the trial combining an OSLO, Clavien-Dindo grade of 2 or higher for early complications and an NCI-CTCAE-v4.0 grade of 3 or higher for late complications. However, results were consistent in the sensitivity analysis considering grade II/2 or higher.

Another limitation was that we were unable to evaluate individual experience level and progression, leading to an evaluation at the center level. In addition, analyses were not corrected for multiple testing, and subgroup analyses should be considered with caution. Finally, we did not use statistical validation procedures allowing evaluation of the predictive value of our models.

## Conclusion

In conclusion, the results of the ROBOGYN-1004 trial suggest that the surgeon must consider different prognostic factors influencing peri- and postoperative morbidity after RL or CL. These includes patient-related factors (performance status or tumor site) that may play a role in enhancing and tailoring preoperative management. The complexity of the surgical procedure must also be taken into consideration, especially when an innovative technology such as RL is introduced in current practices (radical hysterectomy, aortic lymph node dissection).

### Supplementary Information

Below is the link to the electronic supplementary material.

Supplementary file 1 (DOCX 52 kb)
