# Supplementary material for: The Influence of Surgical Complexity and Center Experience on Postoperative Morbidity After Minimally Invasive Surgery in Gynecologic Oncology: Lessons Learned from the ROBOGYN-1004 Trial
Source: Ann Surg Oncol. 2024 Apr 15;31(7):4566–75. doi: 10.1245/s10434-024-15265-1 (PMC11164758; doi:10.1245/s10434-024-15265-1)
Supplement: Supplementary file 1 — (DOCX 52 kb) [file 10434_2024_15265_MOESM1_ESM.docx]

# **SUPPLEMENTARY MATERIAL**

## Table S1: Type of surgery (N=368)

| LND  Hysterectomy | None | Pelvic LND | Aortic LND | Pelvic + Aortic LND | Total |
| --- | --- | --- | --- | --- | --- |
| None | 1 ^(1)^ | 16 | 37 | 14 | 68 |
| Total Hysterectomy (TH) | 122 | 67 | 3 | 14 | 206 |
| Radical Hysterectomy | 37 | 54 | 0 | 3 | 94 |
| Total | 160 | 137 | 40 | 31 | 368 |

LND: lymph node dissection

(1) One patient underwent annexectomy + omentectomy, with neither hysterectomy, nor LND

For the analyis of the type of surgery, we considered four categories combining type of hysterectomy and lymph node dissection as detailed in the table S1:

- The category “TH alone” including 122 patients.
- The category “PeLND+/-TH: Pelvic Lymph Node Dissection with or without Total Hysterectomy” includes 67 patients with PeLND combined with TH, and 16 patients with PeLND without TH, leading to a total of 83 patients.
- The category “AoLND+/-TH: Aortic Lymph Node Dissection, with or without pelvic lymph node dissection, and with or without Total Hysterectomy, but no radical hysterectomy” includes 37 patients with AoLND only, 14 patients with AoLND+PeLND, 3 patients with AoLND+HT and finally 14 with AoLND+PeLND+HT, leading to a total 68 patients.
- The category “RH+/-LND: Radical hysterectomy with or without Lymph Node Dissection” includes 37 patients with RH and no LND, 54 patients with RH and PeLND, and 3 patients with RH combined with PeLND and AoLND, leading to a total of 94 patients.

One patient is included in none of these categories as she underwent annexectomy + omentectomy, with neither hysterectomy, nor LND.

| Group | Type of surgery | N |
| --- | --- | --- |
| TH-alone | Total hysterectomy alone | 122 |
| PeLND+/-TH | Pelvic lymph node dissection with or without TH | 83 |
| AoLND+/-TH | Aortic lymph node dissection with or without TH | 68 |
| RH +/- LND | Radical hysterectomy with or without LND | 94 |

## Table S2: Observed morbidity

|  | **Conventional laparoscopy**  **N=192** | | | | | | | | | | **Robot-assisted laparoscopy**  **N=176** | | | | | | | | | |
| --- | --- | --- | --- | --- | --- | --- | --- | --- | --- | --- | --- | --- | --- | --- | --- | --- | --- | --- | --- | --- |
| **INTRAOPERATIVE MORBIDITY (Oslo classification)** | **No - grade I** | | **Grade II** | | **Grade III** | |  | | **Total**  **Severe (G≥II)** | | **No - grade I** | | **Grade II** | | **Grade III** | |  | | **Total**  **Severe (G≥II)** | |
| **Any type** | 185 | 96% | 7 | 4% | 0 | 0% |  |  | 7 | 4% | 161 | 91% | 13 | 7% | 2 | 1% |  |  | 15 | 9% |
| Hemorrhage | 187 | 97% | 5 | 3% | 0 | 0% |  |  | 5 | 3% | 166 | 94% | 8 | 5% | 2 | 1% |  |  | 10 | 6% |
| Other | 190 | 99% | 2 | 1% | 0 | 0% |  |  | 2 | 1% | 171 | 97% | 5 | 3% | 0 | 0% |  |  | 2 | 1% |
| **EARLY POST-OPERATIVE MORBIDITY (Clavien Dindo classification)** | **No - grade I** | | **Grade II** | | **Grade III** | | **Grade IV** | | **Total**  **Severe (G≥II)** | | **No - grade I** | | **Grade II** | | **Grade III** | | **Grade IV** | | **Total**  **Severe (G≥II)** | |
| **Any type** | 160 | 83% | 25 | 13% | 5 | 3% | 2 | 1% | 32 | 17% | 142 | 81% | 17 | 10% | 14 | 8% | 3 | 2% | 34 | 20% |
| Hemorrhage | 192 | 100% | 0 | 0% | 0 | 0% | 0 | 0% | 0 | 0% | 171 | 97% | 1 | 1% | 3 | 2% | 1 | 1% | 5 | 4% |
| Renal and urinary disorder | 180 | 94% | 11 | 6% | 0 | 0% | 1 | 1% | 12 | 7% | 174 | 99% | 1 | 1% | 1 | 1% | 0 | 0% | 2 | 2% |
| Gastrointestinal disorder | 186 | 97% | 4 | 2% | 0 | 0% | 2 | 1% | 6 | 3% | 170 | 97% | 3 | 2% | 3 | 2% | 0 | 0% | 6 | 4% |
| Infection | 184 | 96% | 6 | 3% | 1 | 1% | 1 | 1% | 8 | 5% | 170 | 97% | 5 | 3% | 1 | 1% | 0 | 0% | 6 | 4% |
| Lymphatic disorder | 186 | 97% | 2 | 1% | 4 | 2% | 0 | 0% | 6 | 3% | 167 | 95% | 3 | 2% | 6 | 3% | 0 | 0% | 9 | 5% |
| Vaginal cuff dehiscence | 0 | 0% | 0 | 0% | 0 | 0% | 0 | 0% | 0 | 0% | 0 | 0% | 0 | 0% | 0 | 0% | 0 | 0% | 0 | 0% |
| Peripheral neurological disorder | 190 | 99% | 2 | 1% | 0 | 0% | 0 | 0% | 2 | 1% | 175 | 99% | 1 | 1% | 0 | 0% | 0 | 0% | 1 | 1% |
| Other | 182 | 95% | 9 | 5% | 1 | 1% | 0 | 0% | 10 | 5% | 164 | 93% | 10 | 6% | 0 | 0% | 2 | 1% | 12 | 7% |
| **LATE POST-OPERATIVE MORBIDITY (NCI CTCAE classification)** | **No - grade 1** | | **Grade 2** | | **Grade 3** | | **Grade 4** | | **Total**  **Severe (G≥3)** | | **No - grade 1** | | **Grade 2** | | **Grade 3** | | **Grade 4** | | **Total**  **Severe (G≥3)** | |
| **Any type** | 175 | 91% | 10 | 5% | 6 | 3% | 1 | 1% | 7 | 4% | 152 | 86% | 16 | 9% | 7 | 4% | 1 | 1% | 8 | 5% |
| Renal and urinary disorder | 189 | 98% | 2 | 1% | 1 | 1% | 0 | 0% | 1 | 1% | 170 | 97% | 4 | 2% | 1 | 1% | 1 | 1% | 2 | 2% |
| Gastrointestinal disorder | 188 | 98% | 3 | 2% | 0 | 0% | 1 | 1% | 1 | 1% | 174 | 99% | 1 | 1% | 1 | 1% | 0 | 0% | 1 | 1% |
| Infection | 189 | 98% | 1 | 1% | 2 | 1% | 0 | 0% | 2 | 1% | 170 | 97% | 3 | 2% | 3 | 2% | 0 | 0% | 3 | 2% |
| Lymphatic disorder | 185 | 96% | 3 | 2% | 4 | 2% | 0 | 0% | 4 | 2% | 168 | 95% | 4 | 2% | 4 | 2% | 0 | 0% | 4 | 2% |
| Vaginal cuff dehiscence | 191 | 99% | 1 | 1% | 0 | 0% | 0 | 0% | 0 | 0% | 174 | 99% | 2 | 1% | 0 | 0% | 0 | 0% | 0 | 0% |
| Other | 185 | 96% | 5 | 3% | 2 | 1% | 0 | 0% | 2 | 1% | 168 | 95% | 5 | 3% | 3 | 2% | 0 | 0% | 3 | 2% |

This table describes the maximal grade observed for perioperative severe morbidity occurred within the 6 months from surgery, according to the type of complication. Results are presented separately for

- intraoperative morbidity graded using Oslo classification,
- early post-operative morbidity defined as complications occurring within 30 days after surgery and graded using Clavien-Dindo classification, and
- late post-operative morbidity defined as complications occurring more than 30 days after surgery and graded using NCI-CTCTAE-v4 classification.

For the main analysis, severe morbidity is defined as grade ≥II of Oslo and Clavien-Dindo classifications and grade ≥3 of NCI-CTCTAE-v4 classification.

## Table S3: Sensitivity analyses of the multivariable model modeling severe perioperative complications, according to the definition of morbidity endpoint (N=368)

|  | **Main analysis**  (GII+/G3+)^1^ 90 events / 368 patients | | | **Sensitivity analysis 1**  (GII+/2+)^2^ 107 events / 368 patients | | | **Sensitivity analysis 2**  (GIII+/3+)^3^ 36 events / 368 patients | | |
| --- | --- | --- | --- | --- | --- | --- | --- | --- | --- |
|  | **OR** | **95%CI** | **P** | **OR** | **95%CI** | **P** | **OR** | **95%CI** | **P** |
| **Treatment arm** |  |  |  |  |  |  |  |  |  |
| Conventional laparoscopy | 1 |  | 0.33 | 1 |  | 0.097 | 1 |  | 0.071 |
| Robot-assisted laparoscopy | 1.28 | 0.78-2.12 |  | 1.51 | 0.93-2.43 |  | 1.95 | 0.94-4.02 |  |
| **WHO Performance status** |  |  |  |  |  |  |  |  |  |
| OR for a 1-point difference | 1.62 | 1.06-2.47 | 0.027 | 1.65 | 0.93-2.44 | 0.019 | 1.09 | 0.57-2.10 | 0.79 |
| **Type of surgery** |  |  | <0.001 |  |  | <0.001 |  |  | 0.29 |
| TH alone | 1 |  |  | 1 |  |  | 1 |  |  |
| PeLND +/- TH | 1.61 | 0.72-3.62 |  | 1.94 | 0.91-4.13 |  | 2.01 | 0.65-6.19 |  |
| AoLND +/- TH | 2.42 | 1.10-5.32 |  | 2.52 | 1.18-5.38 |  | 2.24 | 0.76-6.60 |  |
| RH +/- LND | 4.83 | 2.34-9.98 |  | 5.99 | 2.95-12.1 |  | 2.74 | 0.95-7.90 |  |

OR: Odds ratio; 95%CI: 95% confidence interval

TH=Total Hysterectomy; PeLND=pelvic lymph node dissection; LND=Lymphadenectomy; AoLND=aortic lymph node dissection

(1) In the main analysis, severe perioperative morbidity includes grade II and higher of Oslo classification for intraoperative complications, grade II and higher of Clavien-Dindo classification for early post-operative morbidity (occurring in the 30 days after surgery), and grade 3 and higher of NCI-CTCTAE-v4 for late post-operative morbidity (occurring after 30 days).

(2) In the first sensitivity analysis, we considered as severe morbidity: grade II and higher of Oslo and Clavien-Dindo classifications, and grade 2 and higher of NCI-CTCTAE-v4

(3) In the second sensitivity analysis, we considered as severe morbidity: grade III and higher of Oslo and Clavien-Dindo classifications, and grade 3 and higher of NCI-CTCTAE-v4

## Table S4: Treatment effect (RL versus CL) on the risk of severe perioperative morbidity by sub-group

|  | **Main analysis**  **(GII+/3+)** | | | | **Sensitivity analysis 1**  (GII+/2+)^2^ | | | |
| --- | --- | --- | --- | --- | --- | --- | --- | --- |
| **Subgroup** | **OR_(RL/CL)_** | **95%CI** | | **Interaction  test**  **p-value** | **OR_(RL/CL)_** | **95%CI** | | **Interaction  test**  **p-value** |
| **Age at study entry** |  |  | | 0.75 |  |  |  | 0.93 |
| ≤49 years | 1.12 | 0.44 | 2.90 |  | 1.26 | 0.51 | 3.09 |  |
| 49-58 years | 0.94 | 0.30 | 2.95 |  | 1.32 | 0.44 | 3.94 |  |
| 58-65 years | 2.16 | 0.72 | 6.49 |  | 1.89 | 0.65 | 5.48 |  |
| >65 years | 1.21 | 0.46 | 3.19 |  | 1.69 | 0.66 | 4.32 |  |
| **Body Mass Index at study entry** |  |  |  | 0.96 |  |  |  | 0.88 |
| BMI<30 | 1.28 | 0.70 | 2.35 |  | 1.48 | 0.82 | 2.66 |  |
| BMI≥30 | 1.31 | 0.52 | 3.29 |  | 1.61 | 0.67 | 3.86 |  |
| **WHO performance status  at study entry** |  |  |  | 0.12 |  |  |  | 0.10 |
| WHO 0-1 | 1.44 | 0.86 | 2.41 |  | 1.68 | 1.03 | 2.75 |  |
| WHO 2-3 | 0.14 | 0.01 | 2.42 |  | 0.15 | 0.01 | 2.49 |  |
| **Prior surgery** |  |  |  | 0.29 |  |  |  | 0.42 |
| No prior surgery | 1.16 | 0.68 | 1.98 |  | 1.39 | 0.83 | 2.33 |  |
| Prior surgery | 2.82 | 0.60 | 13.36 |  | 2.57 | 0.65 | 10.2 |  |
| **Prior radiotherapy** |  |  |  | 0.34 |  |  |  | 0.35 |
| No prior radiotherapy | 1.16 | 0.68 | 1.99 |  | 1.37 | 0.81 | 2.31 |  |
| Prior radiotherapy | 2.50 | 0.58 | 10.67 |  | 2.72 | 0.72 | 10.3 |  |
| **Tumor site** |  |  |  | 0.39 |  |  |  | 0.22 |
| Endometrial cancer | 1.00 | 0.48 | 2.10 |  | 1.11 | 0.54 | 2.30 |  |
| Cervical cancer | 1.58 | 0.77 | 3.25 |  | 2.09 | 1.05 | 4.16 |  |
| **Type of surgery** |  |  |  | 0.63 |  |  |  | 0.83 |
| TH alone | 0.77 | 0.27 | 2.23 |  | 1.03 | 0.38 | 2.81 |  |
| PeLND +/- TH | 1.83 | 0.58 | 5.82 |  | 1.97 | 0.70 | 5.59 |  |
| AoLND +/- TH | 1.85 | 0.62 | 5.54 |  | 1.76 | 0.62 | 5.04 |  |
| RH +/- LND | 1.18 | 0.50 | 2.75 |  | 1.48 | 0.64 | 3.44 |  |
| **Center prior RL-experience** |  |  |  | 0.19 |  |  |  | 0.62 |
| <50 RL before start of inclusion | 2.13 | 0.84 | 5.37 |  | 1.81 | 0.74 | 4.44 |  |
| ≥ 50 RL before start of inclusion | 1.02 | 0.56 | 1.85 |  | 1.38 | 0.78 | 2.46 |  |
| **Period of accrual** |  |  |  | 0.52 |  |  |  | 0.36 |
| First half of accrual period | 1.08 | 0.55 | 2.14 |  | 1.20 | 0.62 | 2.30 |  |
| Second half of accrual period | 1.51 | 0.71 | 3.22 |  | 1.89 | 0.91 | 3.92 |  |

TH=Total Hysterectomy; PeLND=pelvic lymph node dissection; LND=Lymphadenectomy; AoLND=aortic lymph node dissection

(1) In the main analysis, severe perioperative morbidity includes grade II and higher of Oslo classification for intraoperative complications, grade II and higher of Clavien-Dindo classification for early post-operative morbidity (occurring in the 30 days after surgery), and grade 3 and higher of NCI-CTCTAE-v4 for late post-operative morbidity (occurring after 30 days).

(2) In the first sensitivity analysis, we considered as severe morbidity: grade II and higher of Oslo and Clavien-Dindo classifications, and grade 2 and higher of NCI-CTCTAE-v4

For each factor successively, we estimated the treatment effect (RL versus CL) in the different subgroups in a multivariable model including WHO performance status, type of surgery, treatment arm, the considered covariate and an interaction term between treatment and the covariate. All models were hierarchical models considering the center as a random effect, except when studying treatment effect according to center prior RL-experience (no random center effect).

For each factor successively, the p-value corresponds to the interaction test of the treatment effect (RL versus CL) by the considered factor.

## Table S5: Treatment effect (RL versus CL) on the risk of severe perioperative morbidity according to the center prior RL-experience and the type of surgery

| **Subgroup** | **CL**  **n/N** | | **RL**  **n/N** | | **OR_(RL/CL)_** | **95% CI** | | **Interaction test P-value** |
| --- | --- | --- | --- | --- | --- | --- | --- | --- |
| **Overall** ^(1)^ | 41/192 | 21% | 49/176 | 28% | 1.28 | 0.78 | 2.12 |  |
| **Type of surgery** ^(2)^ |  |  |  |  |  |  |  | 0.73 |
| Simple (TH alone or PeLND +/- TH) | 16/113 | 14% | 17/92 | 18% | 1.16 | 0.54 | 2.49 |  |
| Difficult (AoLND +/- TH or RH +/- LND) | 25/79 | 32% | 32/83 | 39% | 1.38 | 0.71 | 2.69 |  |
| **Center’s prior RL-experience** ^(3)^ |  |  |  |  |  |  |  | 0.19 |
| <50 RL before start of inclusion | 10/63 | 16% | 19/55 | 35% | 2.13 | 0.84 | 5.37 |  |
| ≥50 RL before start of inclusion | 31/129 | 24% | 30/120 | 25% | 1.02 | 0.56 | 1.85 |  |
|  |  |  |  |  |  |  |  |  |
| **Analysis of interaction  according to the type of surgery** |  |  |  |  |  |  |  |  |
| Simple surgery  (TH alone or PeLND +/- TH) ^(4)^ |  |  |  |  |  |  |  | 0.96 |
| <50 RL before start of inclusion | 5/34 | 15% | 5/21 | 24% | 1.16 | 0.26 | 5.09 |  |
| ≥50 RL before start of inclusion | 11/79 | 14% | 12/70 | 17% | 1.21 | 0.49 | 2.97 |  |
| Difficult surgery  (AoLND +/- TH or RH +/- LND) ^(5)^ |  |  |  |  |  |  |  | 0.07 |
| <50 RL before start of inclusion | 5/29 | 17% | 14/34 | 41% | 3.31 | 1.00 | 11.0 |  |
| ≥50 RL before start of inclusion | 20/50 | 40% | 18/49 | 37% | 0.87 | 0.38 | 1.99 |  |

CL: conventional laparoscopy

RL: robot-assisted laparoscopy

n/N: number of patients experiencing a severe morbidity / number of patients in the subgroup

TH=Total Hysterectomy; PeLND=pelvic lymph node dissection; LND=Lymphadenectomy; AoLND=aortic lymph node dissection; nb evt = number of events (severe morbidity); N= total number of patients

Severe perioperative morbidity includes grade II and higher of Oslo classification for intraoperative complications, grade II and higher of Clavien-Dindo classification for early post-operative morbidity (occurring in the 30 days after surgery), and grade 3 and higher of NCI-CTCTAE-v4 for late post-operative morbidity (occurring after 30 days).

(1) Model 1 includes treatment arm (RL versus CL) + WHO performance status (quantitative value) + type of surgery (TH alone versus PeLND +/- TH versus AoLND +/- TH versus RH +/- LND), considering the center as a random effect

(2) Model 2 includes treatment arm (RL versus CL) + WHO performance status (quantitative value) + type of surgery (simple versus difficult) + treatment arm*type of surgery, considering the center as a random effect

(3) Model 3 includes treatment arm (RL versus CL) + WHO performance status (quantitative value) + type of surgery (TH alone versus PeLND +/- TH versus AoLND +/- TH versus RH +/- LND) + center prior RL-experience (-50 versus +50 robotic acts) + treatment arm*center experience

(4) Model 5 includes treatment arm (RL versus CL) + WHO performance status (quantitative value) + center experience (-50 versus +50 robotic acts) + treatment arm*center experience; considering only patients who had undergone a simple surgery

(5) Model 6 includes treatment arm (RL versus CL) + WHO performance status (quantitative value) + center experience (-50 versus +50 robotic acts) + treatment arm*center experience; considering only patients who had undergone a difficult surgery
